# Unsupervised Flow Cytometry Reveals a Constant Shift Towards Activated CD4 ^+^ T Cell Subsets in APECED

**DOI:** 10.1111/sji.70134

**Published:** 2026-06-26

**Authors:** Joonatan Mattila, Nelli Heikkilä, Iivo Hetemäki, Joonatan Borchers, Outi Mäkitie, Saila Laakso, T Petteri Arstila

**Affiliations:** ^1^ Research Programs Unit, Translational Immunology, and Medicum University of Helsinki Helsinki Finland; ^2^ Children's Hospital, Pediatric Research Center, University of Helsinki and Helsinki University Hospital Helsinki Finland; ^3^ Research Program for Clinical and Molecular Metabolism University of Helsinki Helsinki Finland; ^4^ Folkhälsan Research Center, Helsinki Biomedicum Helsinki Finland; ^5^ Department of Molecular Medicine and Surgery Karolinska Institute and Clinical Genetics, Karolinska University Hospital Stockholm Sweden

**Keywords:** APECED, Treg, unsupervised data analysis

## Abstract

Autoimmune polyendocrinopathy‐candidiasis‐ectodermal dystrophy (APECED) is a rare monogenic autoimmune disease caused by loss‐of‐function mutations in the *Autoimmune regulator* gene and the subsequent impairment of negative selection in the thymus. Previous studies have identified many T cell abnormalities in APECED patients, with increased signs of activation and failure of regulation. These findings, however, have been demonstrated using traditional flow cytometry studies, prone to human misinterpretation of multidimensional data. Here we present an unsupervised flow cytometry analysis of CD4^+^ T cells in 20 APECED patients. Our workflow consists of an optimized sequence of well documented unsupervised analysis algorithms for data quality control, batch correction and clustering with multiple options evaluated. We show the overall increase in T cell activation to extend from the previously reported CD8^+^ compartment to both the T helper and regulatory T cells. We demonstrate the regulatory T cells in APECED to adopt a population distribution dominated by cells of low suppressive potential and present a novel CD45RA^neg^, CCR7^neg^, CD31^high^, CD127^mid^ population unique to APECED. In general, however, we find the population structure of the APECED CD4^+^ T cell compartment largely similar to that of the controls as supported by both our clustering and dimensionality reduction analyses. Our analysis replicates previous findings and provides novel information on APECED while also presenting an optimized unsupervised workflow for flow cytometry analysis in general. Our approach offers multiple advantages over manual flow cytometry analysis with its rigorous data quality control, well‐documented batch correction and optimized automated clustering.

## Graphical abstract

APECED is caused by mutations of *AIRE* that impair negative selection in the human thymus. Using our optimized unsupervised workflow, we reveal that APECED patients show increased activation of T cells and alterations to their Treg pool as well as a novel T cell population not found among healthy controls.

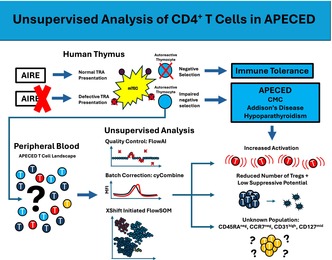

**Abbreviations**
AIRE: autoimmune regulator
APECED: autoimmune polyendocrinopathy‐candidiasis‐ectodermal dystrophy
CM: central memory T cell
CMC: chronic mucocutaneous candidiasis
DCM: dead cell marker
EM: effector memory T cell
MFI: median fluorescence intensity
MNN: mutual nearest neighbours
mTEC: thymic medullary epithelial cell
RTE: recent thymic emigrant T cell
TEMRA: effector memory T cells re‐expressing CD45RA
TfH: T follicular helper
Th: T helper
Treg: T regulatory cell

## 1 Introduction

Autoimmune polyendocrinopathy‐candidiasis‐ectodermal dystrophy (APECED), also called Autoimmune polyendocrine syndrome type 1 (APS1), is a rare but severe recessively inherited monogenic autoimmune disease caused by loss‐of‐function mutations in the *Autoimmune regulator* (*AIRE*) gene [1, 2, 3]. *AIRE* expression is at its highest in the thymic medullary epithelial cells (mTECs) that are crucial in presenting MHC bound autoantigens to the developing thymocytes and inducing negative selection [3, 4, 5]. Based on mouse models, *AIRE* has been deemed a transcriptional master regulator of these tissue‐restricted antigens and APECED the result of their absence in the thymus [6, 7]. *AIRE‐deficient* mice, however, don't reflect the main features of human APECED as they show only mild autoimmune symptoms [8, 9] and lack chronic mucocutaneous candidiasis (CMC), which stands as one of the three main symptoms of human APECED along with hypoparathyroidism and Addison's disease [5, 10].

APECED patients show overall lymphocytosis and multiple abnormalities in their T cell phenotypes, with exaggerated T helper type 1 (Th1) responses and consequently increased interferon‐γ levels as central features of the disease [11, 12, 13]. APECED patients have diminished numbers of CD4^+^ regulatory T cells (Tregs) [14], particularly in early life [15]. This reduction is especially apparent for recent thymic emigrant (RTE) Tregs, whose reduced numbers have been linked to increased attrition [16]. APECED Tregs have previously been reported to express less FOXP3, a key regulator of their suppressive function, and to show impaired suppressive capacity in vitro [14, 16], although both findings have been challenged by a recent study by Sjøgren et al. [17]. According to Heikkilä et al., APECED patients manifest increased proportions of CD4^+^ effector memory (EM) T cells with a decrease in their naïve counterparts, which, however, show abnormal levels of activation [12]. Additionally, APECED patients manifest an expansion of T follicular helper (TfH) cells in the lymph nodes, while the circulating TfH and RTE populations appear diminished [12, 18]. In the CD8^+^ T cell compartment, the reported differences have been even more pronounced, with high expression of both functional and activation markers in APECED [19, 20]. All in all, the known APECED T cell landscape suggests an overall increase in early effector activation with weakened regulatory capabilities [3, 21].

In recent years the manual approach to flow cytometry has grown increasingly challenging, as the number of cell markers evaluated simultaneously has increased drastically with the advancement of flow cytometry hardware. To demonstrate, a full manual analysis of 20 markers would already require inspection of 190 different 2D cytograms with a total of 1,048,576 different marker combinations possible, rendering such an approach virtually impossible. While unsupervised data analysis is common practice in single‐cell RNA sequencing studies, it remains vastly underused in flow cytometry.

Here, we present for the first time an unsupervised flow cytometry analysis of the human T cell pool in APECED, focusing the analysis on CD4^+^ T cells whose compartmentalization into distinct functional populations remains incompletely characterized despite their crucial role in regulating autoimmune events in APECED. By leveraging the unsupervised approach, our workflow offers multiple advantages over the traditional manual methods. First, it introduces a rigorous quality control for the recorded flow cytometry data, greatly reducing possible fluorescence anomalies [22]. Second, it enables well‐documented batch correction, reducing the possibility of misinterpreting technical variations in batched data as biological phenomena [23]. Finally, it decreases the possibility of human misinterpretation by replacing arbitrary and highly individual manual gating strategies with reproducible clustering protocols [24, 25, 26]. Our study provides both novel information about the T cell abnormalities in APECED and a defined, reproducible workflow for unsupervised flow cytometry analysis in general.

## 2 Materials and Methods

### 2.1 Subjects

The study groups consist of 20 APECED patients and 20 healthy controls [27]. The patients and controls were matched by age, with an average age of 46.1 years (range, 23–69 years) in the APECED group and 44.6 years (range, 22–65 years) in the control group. A partial sex match was achieved with 9 men and 11 women in the APECED group and 8 men and 12 women in the control group. 16/20 of the patients were homozygous for the Finnish major mutation R257X while the remaining four showed a heterozygous genotype of R257X compounded with either C311Y (2/20) or a 967–979 deletion (2/20). The patients had an average of 8.3 APECED manifestations, the most common ones being CMC (20/20), Addison's disease (17/20), enamel hypoplasia (16/20), hypoparathyroidism (15/20) and hypogonadism (12/20, 3/9 among men and 9/11 among women). None of the patients had acute infections or were pregnant at the time of sampling. The study was carried out according to the guidelines of the Declaration of Helsinki and approved by the Ethics Committee of the Helsinki University Hospital. A written informed consent was obtained from all participants prior to sample collection. An individual description of each sample donor can be found in Table S1.

### 2.2 Samples and Flow Cytometry

Blood samples from all subjects were taken into BD Vacutainer Heparin Tubes (BD Biosciences, CAT 367880). Plasma was separated from the samples by centrifugation and PBMCs further isolated by Ficoll‐Paque PLUS (GE Life Sciences, CAT 17‐1440‐03) gradient centrifugation. Extracellular staining was performed in a single step after which the cells were permeabilized using the eBioscience FOXP3 permeabilization kit (Thermo Fisher Scientific, CAT 00‐5523‐00) and stained for intracellular markers. All steps were performed according to the manufacturer's instructions. For staining, the following antibodies were used as direct fluorescent conjugates: CD3‐APC‐Cy7, CD4‐BV510, CD45RA‐BV786, CCR7‐BV650, CD31‐Pe‐Cy7, FOXP3‐PE, CD25‐PE‐CF594, CD39‐PerCP‐Cy5, CD127‐Alexa Fluor 700, CTLA‐4‐APC, PD‐1‐BV711, and Ki‐67‐BV421 (Table S2). FITC conjugated CD14, CD19, and dead cell marker (DCM) were used to exclude macrophages, B cells, and dead cells from the analysis, respectively. All the samples were run on LSRFortessa (BD Biosciences) and analysed in the FlowJo analysis software (BD Biosciences). Compensations for spectral overlap were performed using single stained BD Comp beads (BD Biosciences).

### 2.3 Unsupervised Flow Cytometry Analysis

The unsupervised flow cytometry analysis was performed in the FlowJo v10.10 analysis software (BD Biosciences) using plugins generated for unsupervised analysis and data quality evaluation (Data S1). The list of plugins used in the analysis includes: FlowAI, DownSampleV3, cyCombine, XShift, FlowSOM, Euclid, and UMAP [22, 23, 24, 25, 28]. In addition, plugins turned into FlowJo's built‐in features, *t*‐SNE/opt‐SNE and Cluster Explorer were used [29, 30]. Plugins evaluated but not included in the final analysis include: PeacoQC, CytoNorm, Mutual Nearest Neighbours (MNN), Phenograph, and MEM [31, 32, 33, 34].

Our PBMC samples were run in six batches using a single baseline of the flow cytometer LSRFortessa (BD Biosciences). Each batch was designed to contain paired APECED and control samples, all of which were annotated with unique sample identifiers. The collected data was put through rigorous quality control by FlowAI, an algorithm that checks the data for deviations from median flow rate, stability of the fluorescent signal and cells that fall outside the chosen dynamic range removing all cells that fail to meet the selected criteria [22]. On average, FlowAI removed 13.8% of the cells from each sample (range, 1.3%–67.1%), the strict threshold attributable to its requirement for a stable acquisition rate, a feature consistent with manual analysis. Compensation for spectral overlap was applied to the data, which was then manually pre‐gated to singlet, live, CD3^+^ CD4^+^ T cells. A dump of CD14, CD19 and DCM was used to exclude unwanted cells. The pre‐gated data was then randomly downsampled to 25,000 cells per sample to standardize the input of each sample, with the resulting subsets concatenated into a single analysis file containing 1,000,000 CD4^+^ from the 40 samples in total.

The data was then tested for technical batch variations by inspecting a batch annotated t‐SNE plot. As minor batch variation was detected, we used the algorithm cyCombine to perform batch correction and minimize the effect of technical batch variation. Batch correction results were again evaluated with batch annotation overlay on a t‐SNE plot and cyCombine correction plots [23]. As flow cytometry data is fundamentally log‐like, many unsupervised analysis tools require the implementation of careful data scaling prior to use. Thus, the concatenated data were meticulously scaled both before and after batch correction using biexponential scaling and according to the principles of maximal separation and dynamic range usage while preserving the natural shape and relative location of the populations.

The batch corrected data was clustered using an XShift initiated FlowSOM clustering, where XShift was first used to automatically estimate the number of potential cell clusters in the data. Here XShift's initial subsample size was standardized to 25% of the full dataset to stabilize its performance. The clustering results were evaluated by inspecting Euclid derived TaylorIndexes, FlowSOM overlays on t‐SNE plots and UMAPs as well as cluster specific marker expression patterns in FlowJo's Cluster Explorer tool [24, 25, 28, 29]. Repeated FlowSOM runs with slightly differing initiations were performed to guarantee finding the optimal clustering based on the evaluations. The generated T cell clusters were identified using Cluster Explorer and median fluorescence intensity (MFI) tables exported to Microsoft Excel. Finally, the data was visualized with UMAPs optimized for local data preservation. To better represent the unique qualities of the APECED and control populations, clustering and dimensionality reduction were performed separately for both groups.

Statistics were calculated with FlowJo Table Editor and Microsoft Excel. Figures were produced in FlowJo, GraphPad Prism 9 (Dotmatics) and GIMP 3.0.4 (The GIMP Development Team). All analyses were performed using a midrange Lenovo laptop: RAM 32 GB, CPU AMD Ryzen 95900HX, GPU NVIDIA GeForce RTX 3050. This setup facilitated the duration of a single plugin run on 1 million cells to peak at approximately 60 min, with cyCombine, t‐SNE and UMAP taking the longest to finish. Full details of the unsupervised workflow can be found in Data S1 and the detailed description of the batch correction applied in Data S2.

## 3 Results

### 3.1 Unsupervised Analysis Workflow

While multiple different unsupervised analysis algorithms have become readily available through recent years, the challenge of which ones to use for each analysis and how to sequence them remains. Here we present an optimized unsupervised analysis workflow for flow cytometry and use it to analyse the CD4^+^ T cell landscape of 20 APECED patients. The full details of the workflow and analysis can be found in Data S1.

The major unsupervised analysis steps in our workflow can be divided into data quality control, batch correction and clustering. Having run and annotated our samples, we evaluated FlowAI and PeacoQC for quality control. While both algorithms performed well on visual evaluation, the data cleaned by FlowAI underwent more stringent filtering and better reflected the data that would also have passed manual evaluation as FlowAI was set to require a stable flow rate during acquisition, a feature consistent with manual analysis. Thus, FlowAI was selected as the method of choice. As a second step of quality control, we checked the data for technical batch variations by creating a batch annotated t‐SNE plot. As the overlay showed minor but clear batch‐based clustering, we evaluated CytoNorm, MNN and cyCombine algorithms for batch correction [23, 32, 33]. Out of these options CytoNorm was soon ruled out because of its need for additional control samples to function optimally. Between CyCombine and MNN, CyCombine was eventually chosen for the final workflow due to its clear visual output and great performance on large data sets as shown by its ability to produce a smooth distribution of batches on t‐SNE overlays while still retaining the overall cluster structure well (Data S2).

As the overall performance of an unsupervised workflow depends on the quality of the clustering, we next evaluated the main clustering algorithms available in FlowJo, namely Phenograph, FlowSOM, and XShift [24, 25, 34]. As neither Phenograph nor XShift need a user derived estimation of the number of clusters to be searched, they were first compared to each other. Based on both tSNE and UMAP overlays, Phenograph tended to overestimate the cluster number in our samples while XShift, standardized to use a subsample of 25% of the full dataset as its primer, performed better. FlowSOM was then compared to XShift by initiating it with the cluster number estimated by XShift. The results were evaluated using FlowJo's Euclid plugin, which produces a TaylorIndex that assesses the separation of the generated clusters in an N‐dimensional space. The XShift initiated FlowSOM produced consistently higher TaylorIndexes than XShift by itself. Repeated FlowSOM runs revealed that while in most cases the TaylorIndex did not peak at the XShift estimated cluster number, the peak was consistently found in its proximity. We further optimized the clustering by completing several FlowSOM runs in close relation to the TaylorIndex peak and inspecting the resulting FlowSOM clusters on annotated t‐SNE plots and UMAPs as well as marker expression plots in FlowJo's Cluster Explorer, favouring a slight overclustering. This process showed excellent results and led to our final clustering method of an XShift initiated FlowSOM with a thorough Euclid, cluster overlay and marker expression pattern evaluation. The clustered T cell populations were visualized on optimized UMAP overlays and identified with Cluster Explorer and MFI values tabled in Microsoft Excel. Since unsupervised analysis provides no clearcut boundaries but rather a continuum of marker expression levels, the MFI of each individual marker, within every population found, was analysed using a semiquantitative range of negative, low, medium and high expression (Figure S1). As an exception, the manually pre‐gated CD4 was simply marked as CD4^+^.

### 3.2 Distribution of T Helper Cell Clusters Show Small Changes in APECED

**FIGURE 1:**
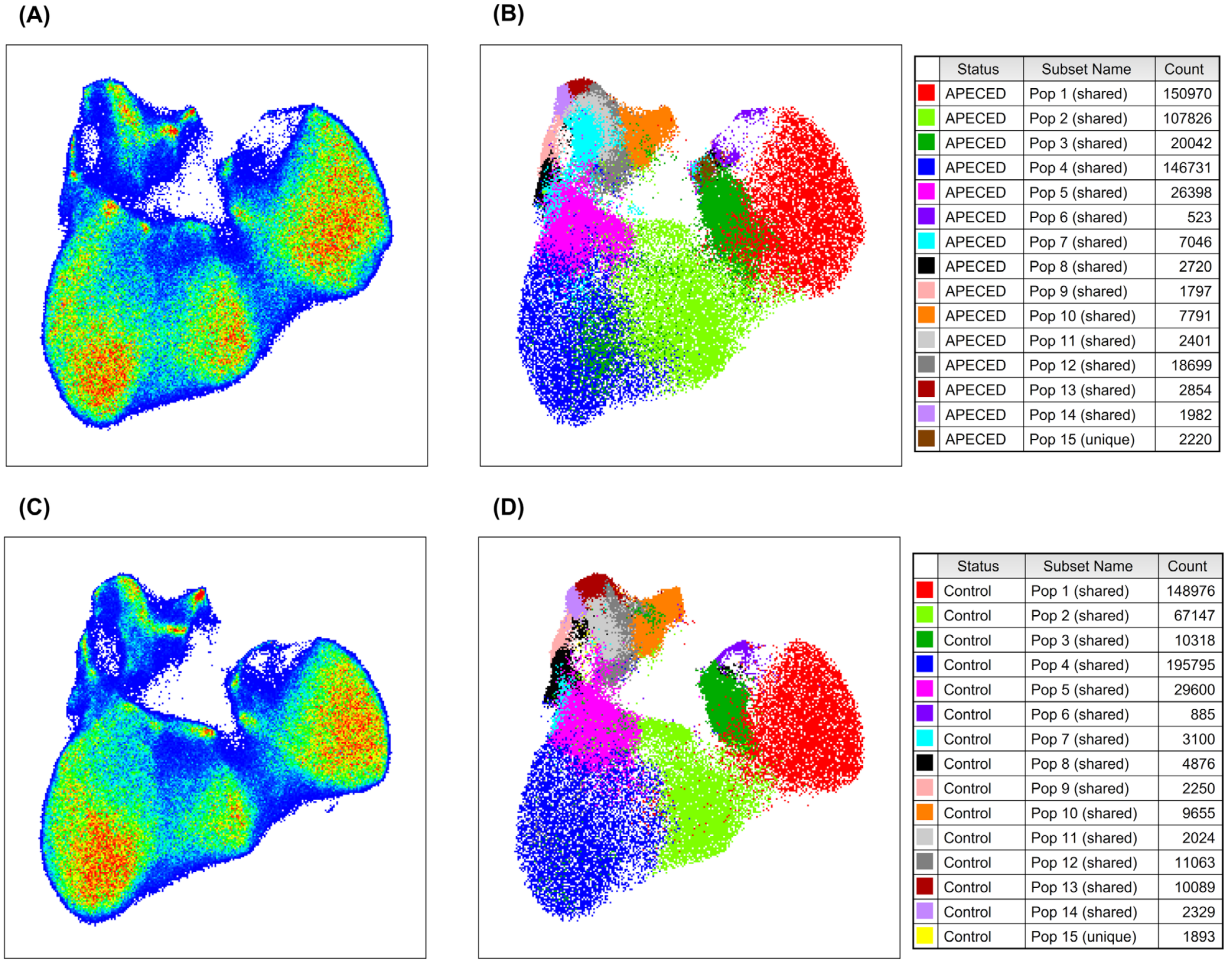
Density and population distribution of CD4^+^ T cells in APECED patients and healthy controls. (A, C) show the cell density distribution UMAPs of 500,000 CD4^+^ T cells from 20 APECED patients and 20 healthy controls, respectively. (B, D) display the population distribution UMAPs of 15 FlowSOM populations generated separately for APECED patients and healthy controls, respectively. Populations 1–14 are shared between APECED patients and healthy controls while population 15 is unique to each study group.

On the UMAP projections, the CD4^+^ T cell compartments of the APECED patients and healthy controls appeared broadly overlapping, with some density differences but no clearly distinct clusters, indicating a largely shared CD4^+^ landscape (Figure 1A,C). Our XShift initiation for clustering produced 11 clusters for both study groups, with our cluster optimization model leading to final FlowSOM runs of 15 clusters per group. Resembling the findings from the UMAP evaluations, 14 of the 15 FlowSOM clusters discovered presented matching identities between the study groups, with these populations ranging through the maturation pathway from RTEs to effector memory T cells re‐expressing CD45RA (TEMRA) (Table 1, shared). Beyond the matching populations, the healthy controls showed one unique Treg population with decreased CD25 expression, while the APECED patients demonstrated an interesting CD45RA^neg^, CCR7^neg^, CD31^high^, CD127^mid^ population with signs of exhaustion (Table 1, unique). These unique populations were relatively small, however, as their fractions comprised no more than 0.4% of the total healthy control and APECED compartments, respectively.

**TABLE 1:** Distribution, marker expression and identities of shared and unique CD4^+^ T cell populations in APECED patients and healthy controls. The top part of the graph represents the shared populations and the bottom the populations unique to either APECED patients or healthy controls. All marker expression levels are shown in median fluorescence intensity (MFI). The colour scale is independently applied to each column and indicates distinct levels of marker expression as follows: white = no expression, blue = low expression, yellow = medium expression, orange = high expression.

Shared	Group	Population	% of parent^a^	CD45RA	CCR7	CD31	FOXP3	CD25	CD39	CD127	CTLA4	PD1	Ki67	Identity	References
Naïve	APECED	Pop 1	30.2	2473	973	981	224	83	37	349	56	60	430	Naïve, Putative RTE	[35]
	Control	Pop 1	29.8	2393	886	1060	203	49	31	372	50	57	351	Naïve, Putative RTE	[35]
	APECED	Pop 2	21.6	1590	952	36	247	115	40	312	70	78	435	Naïve	[36]
	Control	Pop 2	13.4	1758	782	35	224	69	34	285	58	73	337	Naïve	[36]
CM	APECED	Pop 3	4.0	283	904	318	254	112	39	408	63	84	457	Naïve‐CM Transition/CM	[37]
	Control	Pop 3	2.1	118	733	559	252	98	38	454	57	161	391	Naïve‐CM Transition/CM	[37]
	APECED	Pop 4	29.3	109	585	24	291	249	46	625	71	190	481	CM	[36]
	Control	Pop 4	39.2	99	551	21	271	205	40	614	69	164	377	CM	[36]
EM	APECED	Pop 5	5.3	93	282	19	294	78	43	151	80	757	458	EM	[36]
	Control	Pop 5	5.9	81	331	20	277	76	39	147	99	544	389	EM	[36]
TEMRA	APECED	Pop 6	0.1	2557	407	2226	310	91	54	67	252	215	18,798	TEMRA	[38]
	Control	Pop 6	0.2	1617	141	823	252	62	33	29	36	1024	510	TEMRA	[38]
Effector	APECED	Pop 7	1.4	105	433	19	367	125	491	197	171	744	479	Effector/EM, CD39^mid^	[35, 39]
	Control	Pop 7	0.6	83	450	17	258	87	501	266	120	756	431	Effector/EM, CD39^mid^	[35, 39]
	APECED	Pop 8	0.5	112	411	29	333	96	65	153	121	561	14,257	Effector/EM, Ki67^mid^	[35, 39]
	Control	Pop 8	1.0	108	425	26	307	99	67	154	167	664	12,697	Effector/EM, Ki67^mid^	[35, 39]
	APECED	Pop 9	0.4	99	434	24	336	212	143	99	1829	1456	42,755	Effector, Exhausted, Ki67^high^	[35, 39]
	Control	Pop 9	0.5	101	448	20	335	232	112	104	2309	1442	48,704	Effector, Exhausted, Ki67^high^	[35, 39]
Treg	APECED	Pop 10	1.6	1680	658	77	1806	860	75	115	122	70	341	Treg, Naïve, Putative RTE	[40]
	Control	Pop 10	1.9	1575	562	230	1704	867	54	120	75	47	269	Treg, Naïve, Putative RTE	[40]
	APECED	Pop 11	0.5	85	1669	28	3432	1060	106	93	984	158	715	Treg, CM	[41]
	Control	Pop 11	0.4	85	2101	22	3023	821	102	93	921	144	648	Treg, CM	[41]
	APECED	Pop 12	3.7	108	412	24	1998	817	122	98	182	204	390	Treg, Low Potential	[42]
	Control	Pop 12	2.2	117	461	25	1704	687	80	133	135	182	303	Treg, Low Potential	[42]
	APECED	Pop 13	0.6	61	389	30	4887	2335	810	66	804	255	664	Treg, High Potential	[42]
	Control	Pop 13	2.0	50	323	22	3420	1790	459	63	411	223	359	Treg, High Potential	[42]
	APECED	Pop 14	0.4	75	490	27	6903	1531	591	74	2120	306	32,402	Treg, Activated	[43]
	Control	Pop 14	0.5	55	427	29	6309	1734	577	63	1967	269	26,575	Treg, Activated	[43]

Unique	Group	Population	% of parent^a^	CD45RA	CCR7	CD31	FoxP3	CD25	CD39	CD127	CTLA4	PD1	Ki67	Identity	References
Unknown	APECED	Pop 15	0.4	101	350	812	277	65	44	316	69	1096	441	Unknown, CD31^high^, CD127^mid^, PD1^high^	[44]
Treg	Control	Pop 15	0.4	69	329	19	2353	244	624	81	219	428	417	Treg, CD25^low^	[45]

Abbreviations: CM, Central Memory T cell; EM, Effector Memory T cell; RTE, Recent Thymic Emigrant T cell; TEMRA, Effector Memory T cells Re‐expressing CD45RA; Treg, T regulatory cell.

^a^ % of parent = Fraction of all CD4+ cells covered by the population in question.

The naïve and central memory (CM) T helper (Th) compartments dominated the CD4^+^ landscape, with the three largest populations from both the APECED patients and the healthy controls falling under these categories and accounting for combined fractions of 81.1% and 82.4% of all the cells in their respective study groups (Table 1; Figure 1B,D). Both the APECED patients and the healthy controls showed an equal fraction of CD45RA^high^ CCR7^mid^ cells with high CD31 expression (APECED 30.2% vs. Control 29.8%). As CD31 has been described to be increased in CD4^+^ T cells of recent thymic origin [46], this subset was likely to represent RTEs. In contrast, the CD45RA^neg^, CCR7^low^ CM population was substantially larger among healthy controls (APECED 29.4% vs. Control 39.1%) while the APECED patients presented expanded naïve and naïve‐CM transitional cells (CD45RA^mid^, CCR7^mid^, CD31^neg^: APECED 21.6% vs. Control 13.4%; CD45RA^low^, CCR7^mid^, CD31^mid^ APECED 4.0% vs. Control 2.1%) [47]. In the effector‐phenotype Th compartment, the differences in population fractions between the APECED patients and healthy controls were minimal, although the populations appeared slightly larger in the healthy controls. The sole exception to this were the CD39^mid^ effector/EM cells which showed a minor increase in fraction among the APECED patients (APECED 1.4% vs. Control 0.6%).

### 3.3 APECED Tregs Are Skewed Towards Dysfunctional Populations

As shown before by Laakso et al. [16], the APECED patients in our study showed a slightly reduced RTE Treg compartment with the RTE Tregs accounting for 1.6% of all the CD4^+^ cells in APECED patients and 1.9% in healthy controls (Table 1 and Figure 1B,D). Instead, both our study groups demonstrated roughly equal amounts of CM Tregs (APECED 0.5% vs. Control 0.4%). The greatest differences in Treg fractions between study groups were observed when observing populations stratified by suppressive potential. Tregs with low suppressive potential, defined by their lower expression all FOXP3, CD25, CD39, CTLA‐4 and Ki‐67 compared to the other mature Treg populations detected, showed markedly elevated frequencies among the APECED patients (APECED 3.7% vs. Control 2.2%). The APECED Tregs of high suppressive potential instead, appeared with a corresponding decrease in their fraction (APECED 0.6% vs. Control 2.0%). Indeed, although Tregs with low suppressive potential comprised the largest FOXP3^+^ subset in both APECED patients and healthy controls, among the patients they accounted for more than half of all Tregs detected (APECED 55.4% vs. Control 29.9%).

### 3.4 CD4 ^+^ T Cells Shift Towards an Activated State in APECED

The RTE, naïve and CM Th cells of the APECED patients and healthy controls were largely similar in their marker expression levels with no notable differences in either their lineage or functional markers (Table 1, shared). The CD45RA^low^, CCR7^mid^, CD31^mid^ naïve–CM transitional population of the APECED patients, instead, showed a minor elevation in its CD45RA expression compared to the healthy controls (MFI: APECED 283 vs. Control 118). Despite the MFI differences appearing minimal in population‐wise comparisons between the study groups, the APECED naïve and CM compartments collectively showed a constant slight increase in the expression levels of CCR7, FOXP3, CD25, CD39, CTLA‐4, and Ki‐67.

**FIGURE 2:**
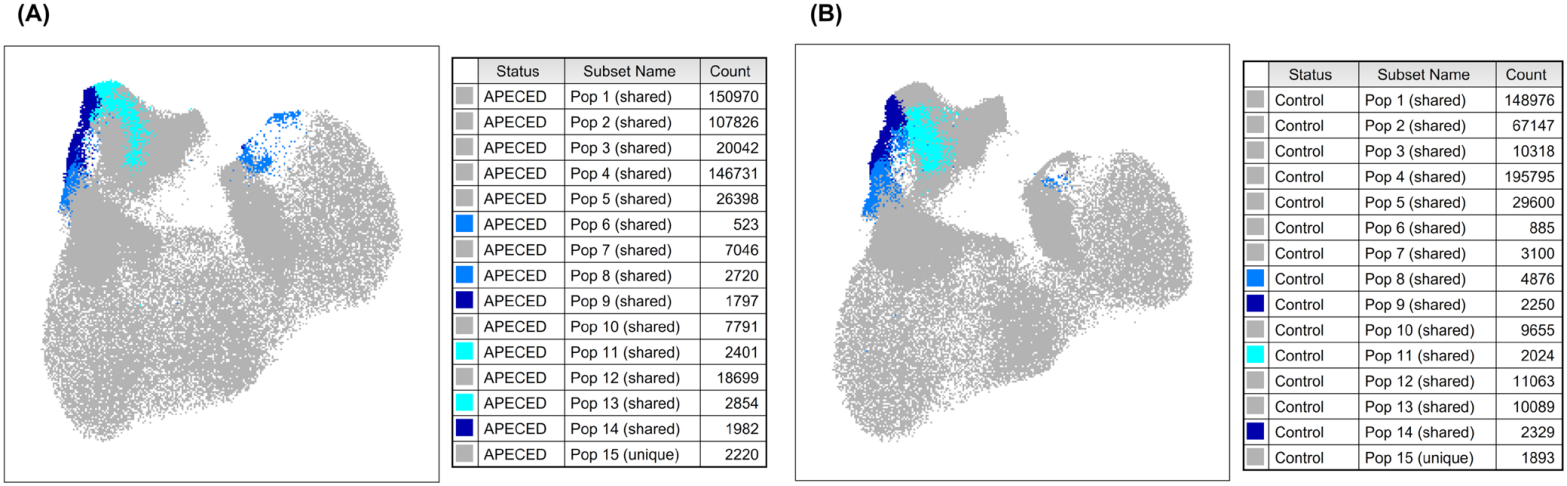
Ki‐67 expression of CD4^+^ T cells in APECED patients and healthy controls. (A, B) show UMAP overlays of the population‐wise Ki‐67 expression of 500,000 CD4^+^ T cells from 20 APECED patients and 20 healthy controls, respectively. Populations 1–14 are shared between APECED patients and healthy controls while population 15 is unique to each study group. Different levels of Ki‐67 expression are indicated with the following colours: Grey = no expression, light blue = low expression, blue = medium expression, dark blue = high expression.

Regarding the Th cells of the effector‐phenotype, the EM and effector populations showed similar marker expression profiles between the APECED patients and healthy controls in pairwise comparisons (Table 1). The minor exception to this was the CD39^mid^ effector/EM cells that showed a slight CD127 decrease in the APECED patients. The CD4^+^ TEMRA cells in our analysis instead demonstrated major differences between the study groups. First, the APECED TEMRA showed a striking increase in their proliferation measured in the levels of Ki‐67 (MFI: APECED 18798 vs. Control 510). Second, this was accompanied by a lack of PD‐1 mediated inhibition (MFI: APECED 215 vs. Control 1024). These notable alterations in the Ki‐67 and PD‐1 levels of the APECED TEMRA marked the two largest population‐wise differences in the study, with the increase in Ki‐67 acting as a hallmark of a constant increase in activation among the effector‐type Th cells in APECED. Additionally, the APECED TEMRA showed a modest increase in their CD45RA as well as minor increases in their CD31 and CTLA‐4, with the latter indicating some retention of inhibition. Overall, the Th cells in APECED showed a slight increase in both their FOXP3 and Ki‐67 levels, detectable in 9/9 and 8/9 of the shared populations respectively (Table 1 and Figure 2).

The expression of CD31 was lower in RTE Tregs from APECED patients than in RTE Tregs from healthy controls (MFI: APECED 77 vs. Control 230) (Table 1). For the most part, the other Treg populations in APECED patients showed higher expression levels in the markers measured. This was true of CD39 in Tregs with low suppressive potential (MFI: APECED 122 vs. Control 80), and of several markers in Tregs with high suppressive potential. APECED Tregs with high potential also expressed higher levels of Ki‐67 (MFI: APECED 664 vs. Control 359). The fully activated Tregs instead appeared similar in phenotype across the groups. As a group, the APECED Treg populations showed minor but uniform increases in multiple markers, most notably FOXP3 and Ki‐67.

## 4 Discussion

Recent flow cytometry studies on both human and mice have shed new light on the function of *AIRE* and the pathogenesis of APECED. The traditional manual analysis tools used in these studies, however, will have to be replaced in the future, as they are becoming insufficient to interpret the data gathered with modern acquisition methods. As a recent effort to modernize the field, Islam et al. utilized broad unsupervised mass spectrometry to reveal an abnormal B cell landscape in APECED patients [48]. Yet, most studies characterizing the overall immune cell, and especially T cell, distributions in APECED have relied on manual flow cytometry analysis, a method that warrants future reassessment.

Here we developed an optimized unsupervised workflow for flow cytometry analysis and utilized it to analyse the phenotypes and distribution of CD4^+^ T cells in 20 APECED patients and 20 healthy controls (Table S1). As recent evidence implicates the exaggerated Th1 responses and subsequent excessive interferon‐γ production as critical, treatable pathogenic mechanisms in APECED [11], we focused our analysis on CD4^+^ Th and Treg cells, whose population distribution and phenotypic heterogeneity remain incompletely characterized. Our workflow allowed us to re‐evaluate results from prior manual flow cytometry studies concerning human APECED but with a scope free of the highly individual, manual gating process known to be prone to human misinterpretation of data [26]. Overall, the T cell marker panel in our study was relatively small, but as such suited well for evaluating our unsupervised workflow against the findings from previous studies analysed with manual methods.

The CD4^+^ APECED T cells in our study showed a notable increase in their overall Ki‐67 levels (Table 1 and Figure 2). This change was already present at the naïve stage, indicating an early shift in the T cell populations towards immune activation. While these results align well with the previous findings on the overactivation of the CD8^+^ as well as the naïve CD4^+^ T cell compartment in APECED [12, 19], we find the consistency and extent of these changes throughout the CD4^+^ compartment remarkable (Table 1). Indeed, the largest MFI difference regarding any marker in our study was the major Ki‐67 increase found among the CD4^+^ APECED TEMRA that also showed a pronounced decrease in their PD‐1 mediated inhibition. Although the population‐wise KI‐67 differences among the other shared populations appeared less pronounced, the exhausted Ki‐67^high^ effectors still represented the only APECED population with decreased Ki‐67 in the study. Remarkably, the most uniform increase in activation was found among the APECED Tregs, with every single Treg population affected. The increased Ki‐67 levels alone, however, do not guarantee elevated suppressive capacity and may instead reflect increased turnover and an exhaustion‐prone phenotype, as suggested by both Laakso et al. and Sjøgren et al. [16, 17].

Our findings showed alterations in Treg cell subset distribution in APECED, with Tregs of low suppressive potential dominating the APECED landscape (Table 1 and Figure 1B,D). In contrast, Tregs with high suppressive potential displayed elevated levels of markers associated with Treg function in the APECED patients. Diverging from some earlier studies, the expression of FOXP3 was higher in APECED patients in our data, a pattern that was visible across both Treg and non‐Treg populations. Our data replicate previous findings of decreased fractions of RTE as well as all Tregs in APECED [14, 16]. While our analysis confirms the low proportions of RTE Tregs in APECED, we don't see a corresponding reduction among the FOXP3^neg^ RTEs. Instead, we demonstrate the CD4^+^ CM population to be strongly diminished in APECED, a defect that seems to be counteracted by the elevated frequencies of the naïve—CM transitional, and especially the naïve T cells among the patients. Whether a similar shift from the traditional RTE and CM populations towards a more transitional state could be seen in Tregs remains to be studied.

Apart from the expected T cell populations, our unsupervised clustering revealed a novel CD4^+^, CD45RA^neg^, CCR7^neg^, CD31^high^, CD127^mid^ Th population among the APECED patients (Table 1). While this expression pattern closely resembles the CD8^+^ EM cells [47], corresponding populations have not been described in detail among the CD4^+^ compartment. Interestingly, this unique population also expressed high levels of the exhaustion marker PD‐1, and since no corresponding population was found among the healthy controls, it may be involved in the APECED disease process. It is, however, good to notice that this population was relatively small as it only covered 0.4% of the APECED T cells analysed.

## 5 Conclusions

Here, using our unsupervised workflow, we confirm the previously reported increased activation state of the naïve CD4^+^ T cells in APECED and extend this elevated activation to cover the mature Th and Treg cells as well. Although we demonstrate the phenotypes of individual APECED Tregs to appear relatively normal or in some respects of increased capability, we reveal the APECED Tregs to have a highly defective population distribution, suggesting errors in peripheral Treg recruitment. Additionally, we report a previously unknown CD4^+^, CD45RA^neg^, CCR7^neg^, CD31^high^, CD127^mid^ T cell population among the APECED patients, the functional properties and the origin of which remain to be studied.

## Author Contributions

**Joonatan Mattila:** conceptualization (equal); data curation (equal); formal analysis (lead); investigation (supporting); methodology (lead); software (lead); validation (lead); visualization (lead); funding acquisition (supporting); writing – original draft preparation (lead); writing – review and editing (equal). **Nelli Heikkilä:** conceptualization (equal); data curation (equal); formal analysis (equal); investigation (equal); writing – review and editing (equal). **Iivo Hetemäki:** conceptualization (equal); data curation (equal); formal analysis (equal); investigation (equal); writing – review and editing (equal). **Joonatan Borchers:** investigation (supporting); writing – review and editing (equal). **Outi Mäkitie:** funding acquisition (equal); supervision (equal); resources (equal); writing – review and editing (equal). **Saila Laakso:** data curation (equal); investigation (equal); funding acquisition (equal); project administration (equal); writing – review and editing (equal). **T Petteri Arstila:** conceptualization (equal); funding acquisition (lead); project administration (lead); supervision (lead); resources (lead); writing – review and editing (equal).

## Funding

This study was funded by impartial foundations and research programs; we did not receive any corporate funding. Grants from Helsinki University Hospital, Päivikki and Sakari Sohlberg Foundation, Finnish Foundation for Paediatric Research, Research Council of Finland, Sigrid Jusélius Foundation, Finnish Medical Foundation (grants no. 4145 and 6558) and the MD PhD program of the Faculty of Medicine of the University of Helsinki were used to pay for human resources. Grants from Helsinki University Hospital, Sigrid Jusélius Foundation and Novo Nordisk Foundation facilitated the costs of study visits. A grant from Liv och Hälsa Foundation facilitated material costs for the study. Open access funded by Helsinki University Library.

## Ethics Statement

This study was approved by the Ethics Committee of the Helsinki University Hospital and carried out according to the guidelines of the Declaration of Helsinki. A written informed consent was obtained from all participants prior to sample collection. No potentially identifiable images or data are presented in this study.

## Conflicts of Interest

The authors declare no conflicts of interest.

## Supporting information


**Table S1:** Description of the sample donors. Batch distribution, sex and age are shown for both the APECED patients and the healthy controls. Age is presented in categories to protect patient anonymity. For each APECED patient, the presence of the most frequent and total disease components is also listed. CMC, Chronic Mucocutaneous Candidiasis.

**Table S2:** Detailed description of the antibodies and their direct fluorescent conjugates.

**Data S1:** Detailed description of the unsupervised analysis workflow.

**Data S2:** Batch correction documentation.

**Figure S1:** Cell count, frequency of parent and median fluorescence intensity (MFI) per marker for each CD4^+^ T cell population detected. For each panel, the corresponding values are plotted in descending order for APECED patients and healthy controls separately, to better represent the population hierarchy relative to the markers analysed.

## Data Availability

The data that support the findings of this study are available on request from the corresponding author. The data are not publicly available due to privacy or ethical restrictions.
