# Supplementary material for: Unsupervised Flow Cytometry Reveals a Constant Shift Towards Activated CD4 + T Cell Subsets in APECED
Source: Scand J Immunol. 2026 Jun 26;104(1):e70134. doi: 10.1111/sji.70134 (PMC13307629; doi:10.1111/sji.70134)
Supplement: Supplementary file 3 — Data S1: Detailed description of the unsupervised analysis workflow. [file SJI-104-e70134-s002.docx]

**Supplement 3**

Here we present a detailed description of the unsupervised analysis workflow used.

**Unsupervised analysis in flow cytometry**

While unsupervised data analysis is a commonly implemented tool in single cell RNA sequencing studies, it remains vastly underused in flow cytometry studies where traditional workflows with manual, user driven population gating still dominate. These manual approaches, however, are becoming extremely laborious and prone to human error and misinterpretation as the number of cell markers in flow cytometry studies gradually increases with the rapid advancement of the flow cytometry hardware. The problems of a manual flow cytometry analysis become evident when inspecting two different flow cytometry panels, one with 10 and another with 20 markers. While with the panel of 10 markers, every marker can be plotted against every other marker using 45 different 2D cytograms, the panel of 20 markers already requires a massive 190 2D cytograms to achieve full coverage the traditional way. In terms of possible marker combinations in total, the 10-marker panel presents 1023 and the 20-marker panel 1048576 different options. This increase makes the manual approach virtually impossible both in terms of human resources needed and because the human brain cannot grasp the marker interactions that take place across hundreds of thousands to millions of simultaneous planes. Thus, a reliable, reproducible unsupervised approach is imperative. In recent years, several excellent analysis tools have been developed with multiple different options to choose from for each step of the analysis. Yet the problem of how to select the optimal tools combination for a reproducible workflow remains. Here we have analyzed 20 APECED patients and 20 healthy controls with an optimized unsupervised analysis workflow based on available analysis tool plugins on FlowJo (BD Biosciences).

**Data acquisition and annotation**

As acquiring every patient or even control sample simultaneously is often impossible, the individually acquired PBMC samples were first frozen in -70°C. The samples were then thawed and stained, and the flow cytometry run in a total of 6 batches of 4 or 8 samples to reduce mechanical variance resulting from possible day-to-day alterations in the staining and running processes. Each batch was designed to contain an equal number of APECED and control samples with each APECED sample having an age and sex matched control sample within the same batch when possible. The carefully planned batch allocation was crucial to ensure that a possible batch correction would not hamper the detection of biological variance between the study groups but rather eliminate mechanical variation between the batches. All batches were run between 1/2017 and 5/2017 using the same baseline of the running machinery LSRFortessa (BD Biosciences). As our approach requires the later concatenation of all samples, a thorough annotation was applied to the data by giving each sample a unique sample identifier as well as identifiers for batch and disease status (APECED/control).

**Quality control**

Next, each sample was evaluated for data quality. For this purpose, we chose the algorithm FlowAI that checks the data for deviations from median flow rate, stability of the fluorescent signal and cells that fall outside the chosen dynamic range, removing the cells not meeting the selected criteria from the analysis (1). FlowAI was run including all the fluorescent parameters in the quality assessment and using the default quality criteria (Second fraction FR 0.100, Alpha FR 0.0100, Maximum changepoints 3, Changepoint penalty 200) as our repeated testing revealed no benefit from altering these. We also compared FlowAI against another well-established quality control algorithm PeacoQC (2). In our analysis FlowAI removed on average 13.8 % of the cells from each sample (range, 1.3 – 67.1 %), whereas PeacoQC only removed 4.3 % (range, 0 – 20,4 %). Both algorithms performed generally well upon a visual inspection of their quality control output, with PeacoQC less effective in its removal of data that would have failed manual inspection. In most cases, FlowAI removed the same portions of the data as PeacoQC but with a wider margin, a feature attributable to its requirement for a stable flow rate during data acquisition. The strict FlowAI was thus selected as the quality control method of choice, ensuring the stable, good quality of our data. The resulting increase in data loss to quality control was evaluated inconsequential as an eventual down sampling was ultimately required due to computational limitations. It is good to notice that FlowAI was run before and PeacoQC after compensation for spectral overlap as recommended by FlowJo plugin documentation.

**Manual pre-gating**

Even if our workflow is unsupervised in nature, some amount of pre-gating is necessary to focus the analysis so that computational power is saved, and possible significant findings aren’t lost to noise arising from debris and unwanted cell types. This pre-gating process, however, should be restricted to the dimensional FSC / SSC parameters and well-defined lineage markers with clear division between positive and negative populations to minimize potential bias from human decision making and assumptions made on prior knowledge. Thus, after the quality control with FlowAI, we manually removed debris and unwanted cells like macrophages, B-cells and dead cells from the analysis. We then focused the data on T cells by only including CD3^+^ cells and further refined the data selection by strictly gating to CD4^+^ cells only. Compensation for spectral overlap was applied in cohesion with the pre-gating and based on single stained BD Comp beads (BD Biosciences). Initial data scaling was also performed during this step using the transform function of FlowJo. Finally, each pre-gated sample was randomly downsampled to 25 000 cells using the DownSampleV3 plugin, and the resulting datasets were concatenated into a single analysis file of 1 million CD4^+^ T cells, ensuring equal input of cells from each sample.

**Batch correction and data scaling**

To evaluate whether there was technical variance between the batches, a t-SNE plot was generated based on the concatenated data and overlayed with batch annotation (3). As this showed minor but clear batch-based clustering, a batch correction was applied to the data. For batch correction 3 different methods were evaluated: cyCombine, Mutual Nearest Neighbors (MNN) and CytoNorm (4–6). CytoNorm was rapidly excluded as it requires separate batch correction controls to function to its full potential. While MNN and cyCombine produced similar results on small test files, cyCombine outperformed MNN on sample sizes exceeding 400 000 cells, producing smoother distribution of batches on t-SNE overlays, yet still retaining the overall cluster structure well. On top of the better performance in testing, FlowJo’s cyCombine plugin provides the user with a histogram-based visualization of the correction performed per batch and marker, a feature which the MNN plugin lacks. Thus, cyCombine was selected as the batch correction method of choice. All batch correction results were evaluated on batch overlays on t-SNE as well as cyCombine correction plots, both of which showed excellent results when using cyCombine.

As flow cytometry data is fundamentally log-like, most batch correction, dimensionality reduction and clustering plugins on FlowJo use scaled data as input as also recommended by other industry staples like OMIQ (Dotmatics). Thus, meticulous biexponential scaling was performed on the concatenated data prior to the batch correction, using FlowJo’s transform function and following three main principles. First, the unused dynamic range was minimized. Second, the separation of positive and negative signals for each marker was increased to the maximum, and third, all this was done while maintaining the natural shape and relative location of the biological populations. As the batch correction plugins tended to alter the scaling of the data, the scaling parameters were thoroughly revaluated after the batch correction step. All the used scaling parameters from both before and after the batch correction step were recorded to enhance reproducibility of the analysis.

**Unsupervised clustering**

With the data quality evaluated and the batch correction applied, unsupervised clustering was introduced to the data. To better represent the individual qualities and possible unique populations of the APECED patients and the healthy controls, the clustering was performed separately for both groups. For clustering Phenograph, FlowSOM and XShift were evaluated (7–9). Phenograph and XShift automatically estimate the number of clusters in the data whereas FlowSOM requires this information as an input from the user. Both approaches have their advantages. As Phenograph and XShift offered similar functionality, they were first compared with each other. Based on cluster overlays on t-SNE and UMAP, Phenograph tended to noticeably overestimate the number of clusters with our sample size of 500 000 cells whereas XShift’s estimation was found closer to optimal already on default settings. However, as FlowJo’s XShift plugin uses by default a fixed subsample of 100 000 cells to initiate its clustering process irrespective of the input dataset's total cell count, we noticed that default XShift runs on smaller datasets paradoxically tended to result in increased cluster numbers. Thus, we normalized the cell count of the initial subsample to always cover 25% of the full input dataset which improved the stability of the estimation.

We then compared XShift with FlowSOM by initiating FlowSOM with XShift’s estimate for cluster number and evaluating the clustering results from both algorithms with FlowJo’s Euclid plugin that applies two distinct checks to the clustering. First, Euclid produces a “DipTest” based ClustRCheck modality score that measures the presence of deeper subpopulations within each cluster (10). Second and foremost, Euclid calculates a Stain-Index-like TaylorIndex that checks the separation of all input clusters in an N-Dimensional space and can be used to evaluate the goodness of clustering results. In our analysis the XShift initiated FlowSOM produced consistently higher TaylorIndexes than XShift by itself. Repeated FlowSOM runs over a range on cluster numbers close to the original XShift estimation revealed that while in most cases the TaylorIndex did not peak at the original XShift estimation, the peak was consistently found in its proximity. To fight the potential loss of small unique populations to possible underclustering, we then evaluated FlowSOM runs with a cluster number 0-5 clusters higher than the one producing the optimal TaylorIndex by inspecting cluster overlays on both t-SNE and UMAP and marker expression patterns of each cluster in FlowJo’s Cluster Explorer. A slight overclustering was favored in the evaluation to preserve the appearance of the rarer populations. Thus, our final clustering method consists of an XShift initiated FlowSOM with a thorough Euclid, cluster overlay and marker expression pattern evaluation.

**Visualization and population identification**

After clustering, both t-SNE and UMAP were evaluated for final visualization. For both dimensionality reduction methods multiple settings were tested considering the large size of the datasets. In general, UMAP produced a more comprehensive clustering with a better match to the populations from the optimized clustering and was selected as the method of choice. All UMAPs were generated with the FlowJo UMAP plugin using a nearest neighbors value of 25 and a distance value of 0,25. The nearest neighbors value was set lower than the FlowJo default of 50 to favor local data preservation and the distance value higher than the FlowJo default of 0,15 to prevent data clumping with the increased spread of the local data.

To identify the clusters, all median fluorescence intensity values (MFI) categorized by cluster and marker were exported from FlowJo to Microsoft Excel. The marker-wise MFIs were then ranked on a semiquantitative range from negative to high using line plots to better distinguish turning points between the ranks. Finally, the ranked marker expression patterns were analyzed and compared to existing literature to identify the clusters found. For cluster identification, we also tested the novel Marker Enrichment Modeling algorithm (MEM), which automatically assesses the marker expression patterns of each cluster and ranks the cluster-wise MFIs on a scale of 1-10 by comparing them to an automatically or manually selected reference population (11). This approach was abandoned, however, as bright markers like Ki-67 tended to set off the MEM scaling which in turn resulted in dimmed separation of the of the populations by other markers in the analysis.

**References**

1. Monaco G, Chen H, Poidinger M, Chen J, de Magalhães JP, Larbi A. flowAI: automatic and interactive anomaly discerning tools for flow cytometry data. Bioinformatics. 2016;32:2473–2480. doi: 10.1093/bioinformatics/btw191.

2. Emmaneel A, Quintelier K, Sichien D, Rybakowska P, Marañón C, Alarcón-Riquelme ME, Van Isterdael G, Van Gassen S, Saeys Y. PeacoQC: Peak-based selection of high quality cytometry data. Cytometry A. 2022;101:325–338. doi: 10.1002/cyto.a.24501.

3. Laurens van der Maaten, Geoffrey Hinton. Visualizing Data using t-SNE. J Mach Learn Res. 2008;9:2579–2605.

4. Pedersen CB, Dam SH, Barnkob MB, Leipold MD, Purroy N, Rassenti LZ, Kipps TJ, Nguyen J, Lederer JA, Gohil SH, et al. cyCombine allows for robust integration of single-cell cytometry datasets within and across technologies. Nat Commun. 2022;13:1698. doi: 10.1038/s41467-022-29383-5.

5. Van Gassen S, Gaudilliere B, Angst MS, Saeys Y, Aghaeepour N. CytoNorm: A Normalization Algorithm for Cytometry Data. Cytometry A. 2020;97:268–278. doi: 10.1002/cyto.a.23904.

6. Haghverdi L, Lun ATL, Morgan MD, Marioni JC. Batch effects in single-cell RNA-sequencing data are corrected by matching mutual nearest neighbors. Nat Biotechnol. 2018;36:421–427. doi: 10.1038/nbt.4091.

7. Van Gassen S, Callebaut B, Van Helden MJ, Lambrecht BN, Demeester P, Dhaene T, Saeys Y. FlowSOM: Using self-organizing maps for visualization and interpretation of cytometry data. Cytometry A. 2015;87:636–645. doi: 10.1002/cyto.a.22625.

8. Samusik N, Good Z, Spitzer MH, Davis KL, Nolan GP. Automated mapping of phenotype space with single-cell data. Nat Methods. 2016;13:493–496. doi: 10.1038/nmeth.3863.

9. Levine JH, Simonds EF, Bendall SC, Davis KL, Amir ED, Tadmor MD, Litvin O, Fienberg HG, Jager A, Zunder ER, et al. Data-Driven Phenotypic Dissection of AML Reveals Progenitor-like Cells that Correlate with Prognosis. Cell. 2015;162:184–197. doi: 10.1016/j.cell.2015.05.047.

10. Freeman JB, Dale R. Assessing bimodality to detect the presence of a dual cognitive process. Behav Res Methods. 2013;45:83–97. doi: 10.3758/s13428-012-0225-x.

11. Diggins KE, Gandelman JS, Roe CE, Irish JM. Generating Quantitative Cell Identity Labels with Marker Enrichment Modeling (MEM). Curr Protoc Cytom. 2018;83:10.21.1-10.21.28. doi: 10.1002/cpcy.34.
