# Supplementary material for: Unsupervised Flow Cytometry Reveals a Constant Shift Towards Activated CD4 + T Cell Subsets in APECED
Source: Scand J Immunol. 2026 Jun 26;104(1):e70134. doi: 10.1111/sji.70134 (PMC13307629; doi:10.1111/sji.70134)
Supplement: Supplementary file 4 — Data S2: Batch correction documentation. [file SJI-104-e70134-s005.docx]

**Supplement 4**

Here we present a detailed description of the batches and batch correction applied. All our samples were stained, and the flow cytometry ran in six separate batches of 4 or 8 samples to reduce mechanical variance resulting from possible day-to-day alterations in the staining and running processes. Each batch was designed to contain an equal number of APECED and control samples with each APECED sample having an age and sex matched control sample within the same batch when possible. As batch variation is a well-known source of technical bias, we produced a tSNE plot with the batch annotation overlayed to evaluate the amount of possible batch variation in our data. A subtle but clear batch variation was detected among the data, shown as the uneven distribution of the standardized batches when overlayed on the tSNE plot (Figure 1). We thus applied a well-documented cyCombine (1) batch correction to our data and evaluated its performance on a second batch annotation overlayed tSNE plot as well as marker correction plots produced by cyCombine. The tSNE overlay showed excellent normalization of the technical batch variation with homogenous batch distribution (Figure 1). Importantly the general morphology and cluster structure of the tSNE plot were well retained, indicating minor effect of the batch correction to the biological variance of the data. These results were confirmed by the cyCombine correction plots that showed uniform batch-wise histogram placing and shape for the batch corrected marker expression values while achieving this with only minor alterations to the raw data (Figure 2).


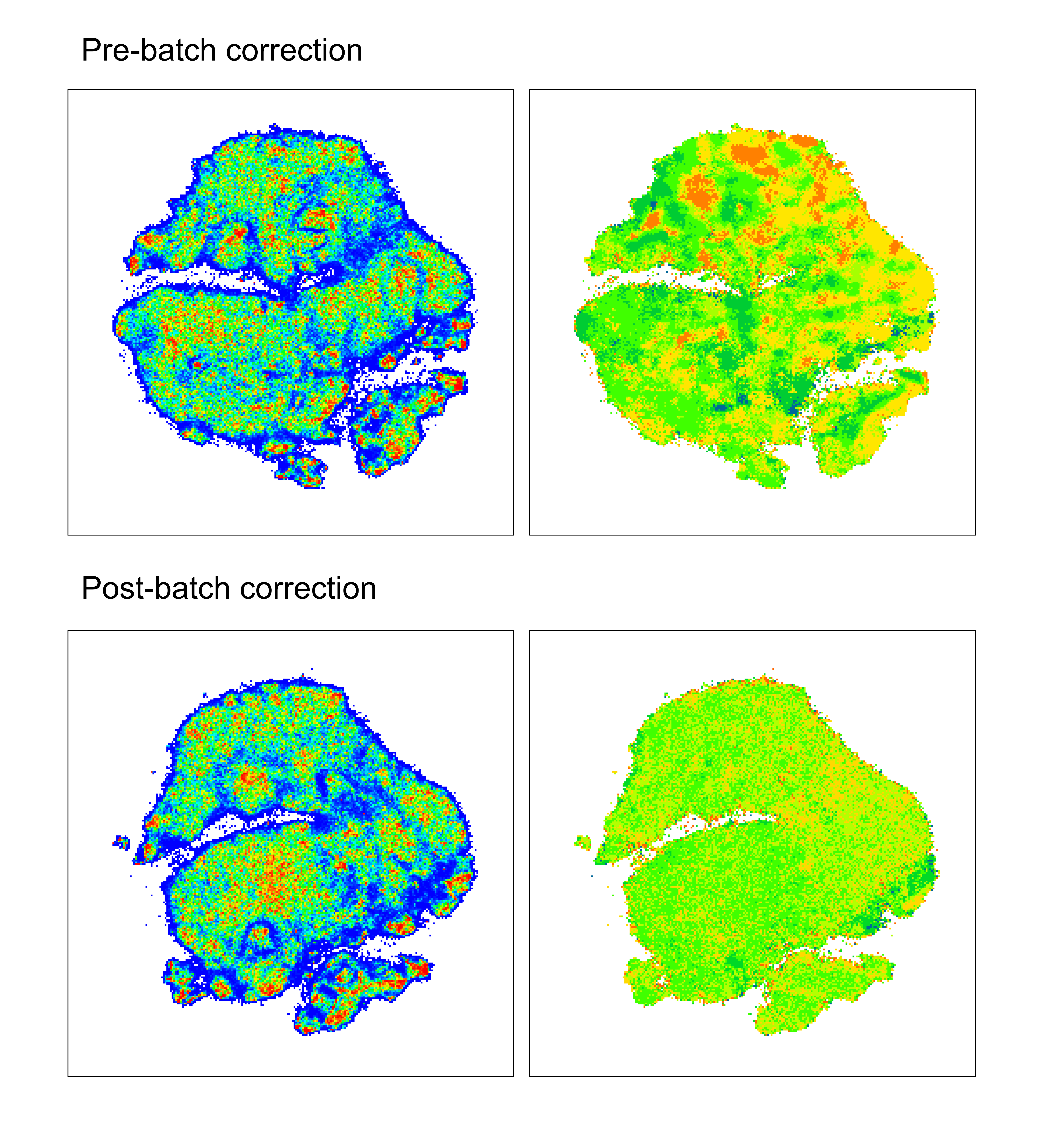


**Figure 1.** cyCombine batch correction of concatenated CD4^+^ T cells from 20 APECED patients and 20 healthy controls. The left-hand panels show the cell density distribution t-SNE plots pre- and post-batch correction while the right-hand panels show the same t-SNE plots with batch annotation overlay applied.


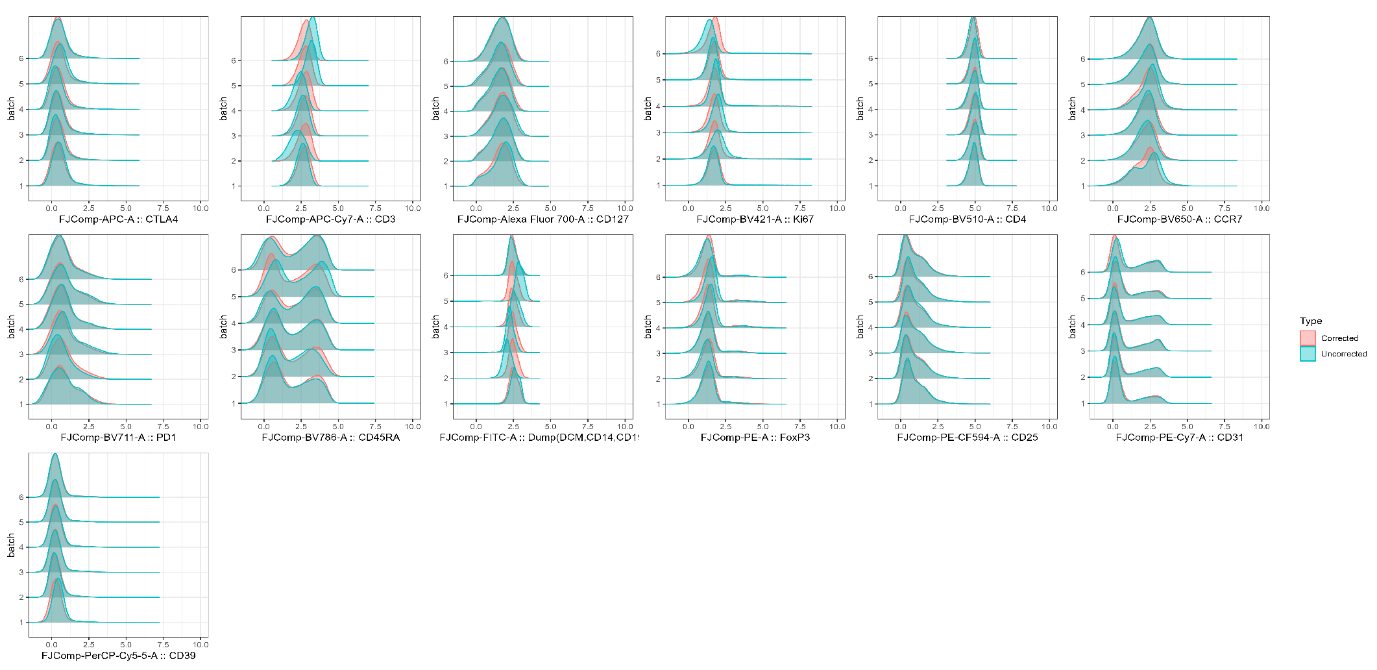


**Figure 2.** cyCombine correction plot of concatenated CD4^+^ T cells from 20 APECED patients and 20 healthy controls. The histograms show the pre- and post-batch correction marker expression level and distribution for each marker and batch individually.

**References**

1. Pedersen CB, Dam SH, Barnkob MB, Leipold MD, Purroy N, Rassenti LZ, Kipps TJ, Nguyen J, Lederer JA, Gohil SH, et al. cyCombine allows for robust integration of single-cell cytometry datasets within and across technologies. Nat Commun. 2022;13:1698. doi: 10.1038/s41467-022-29383-5.
