# Supplementary figures and images for: Unsupervised Flow Cytometry Reveals a Constant Shift Towards Activated CD4 + T Cell Subsets in APECED
Source: Scand J Immunol. 2026 Jun 26;104(1):e70134. doi: 10.1111/sji.70134 (PMC13307629; doi:10.1111/sji.70134)

# CD4<sup>+</sup> T cell populations

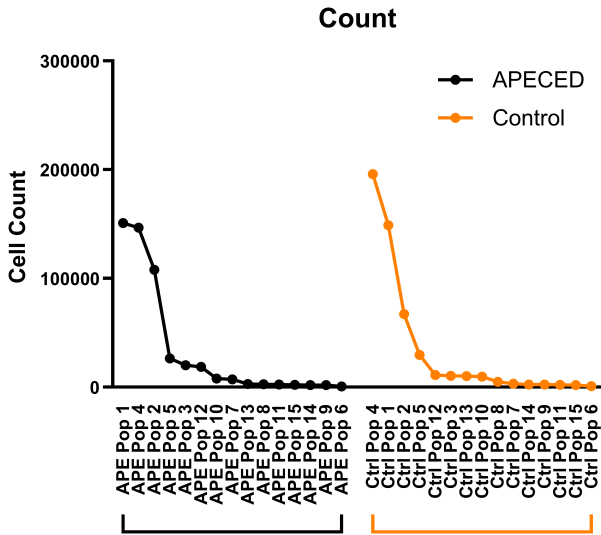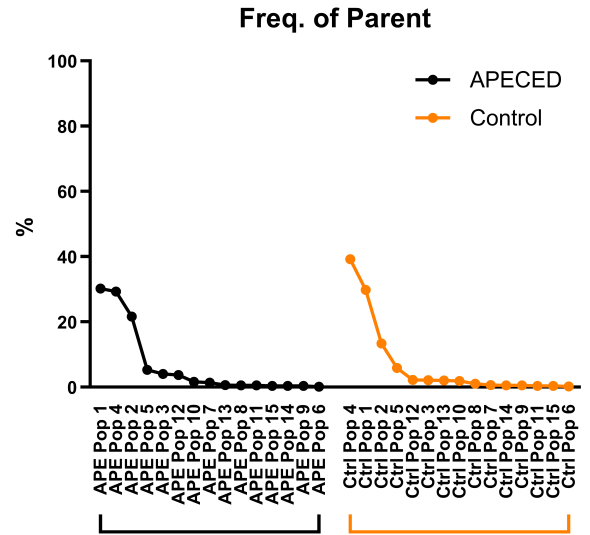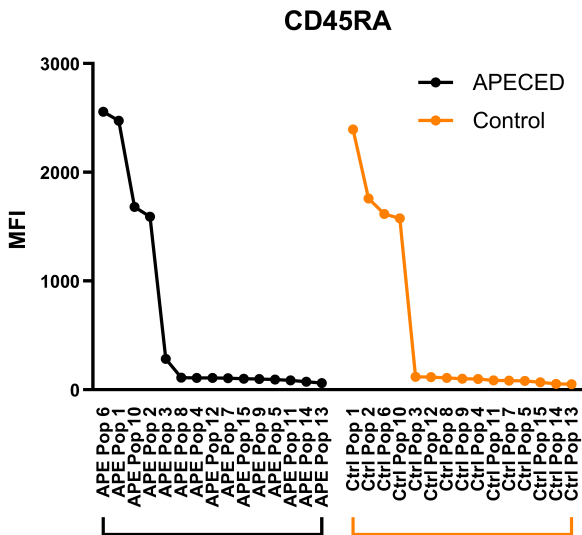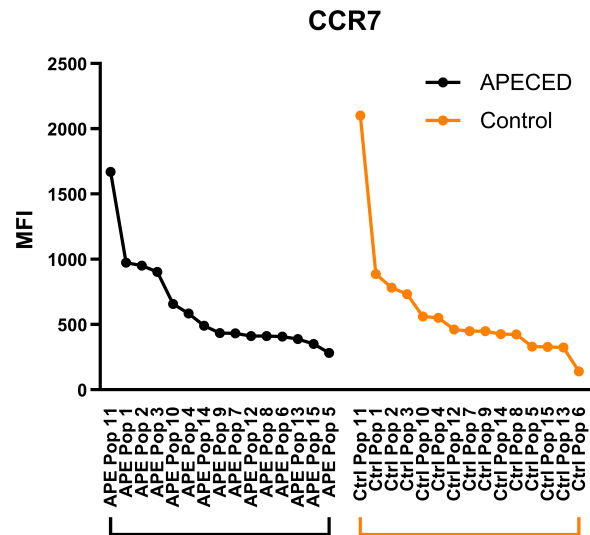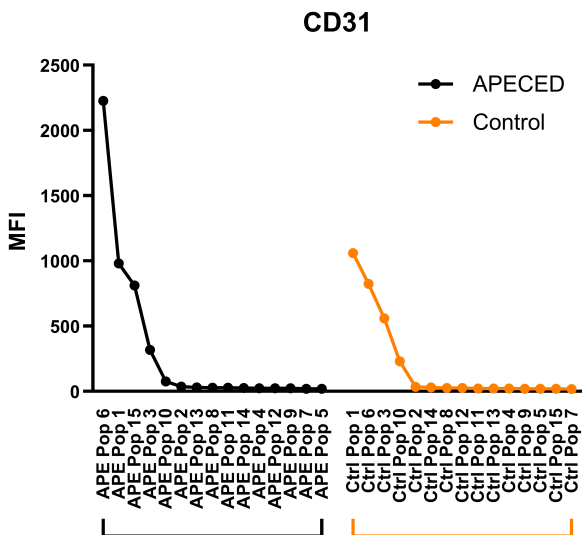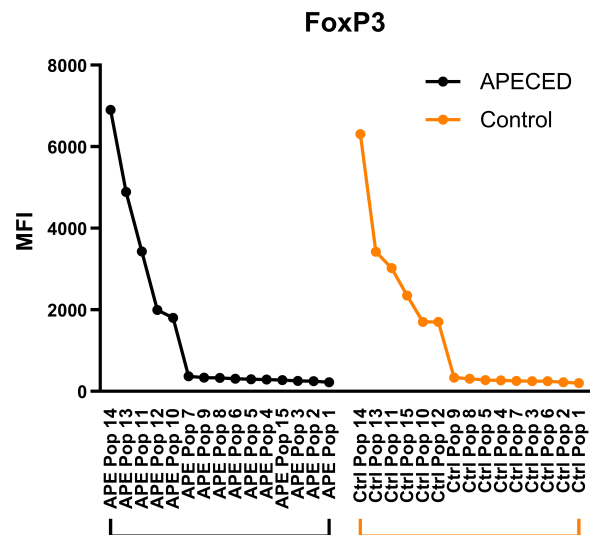

CD25

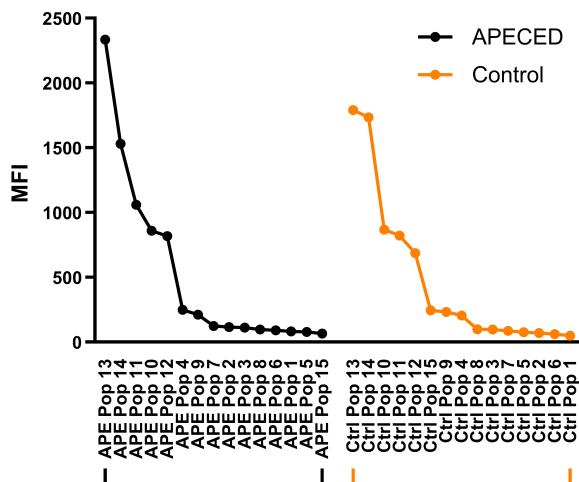

CD39

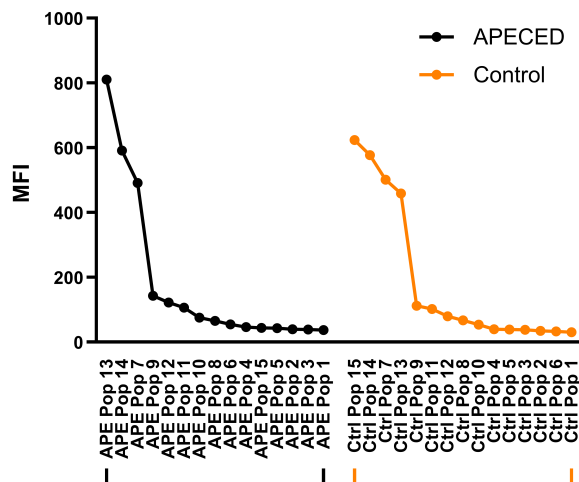

CD127

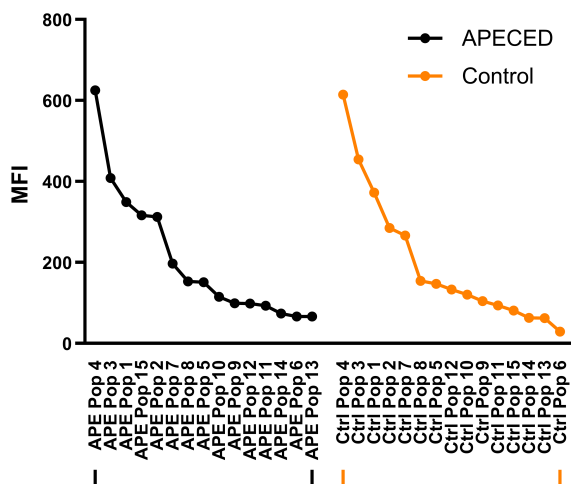

CTLA-4

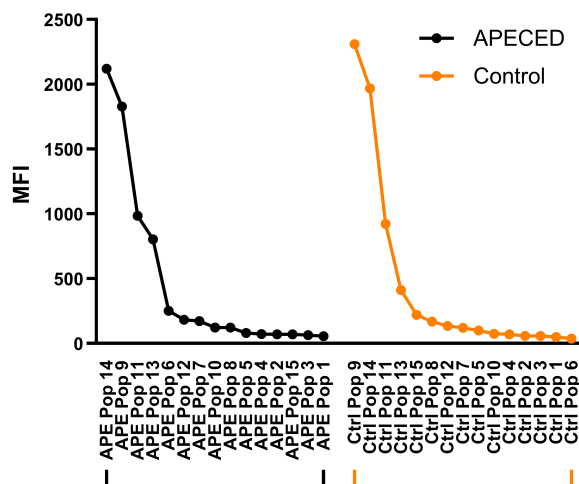

PD-1

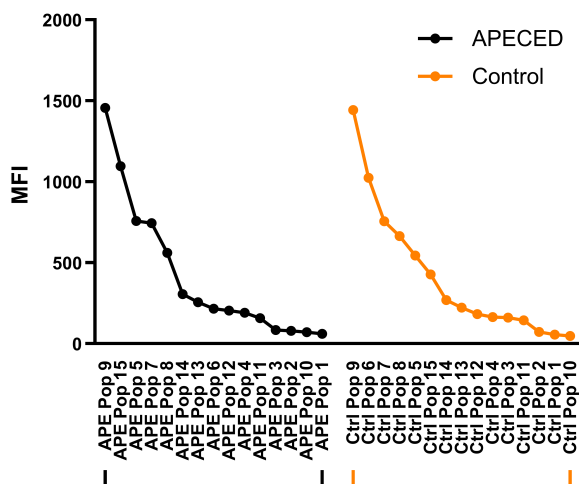

Ki-67

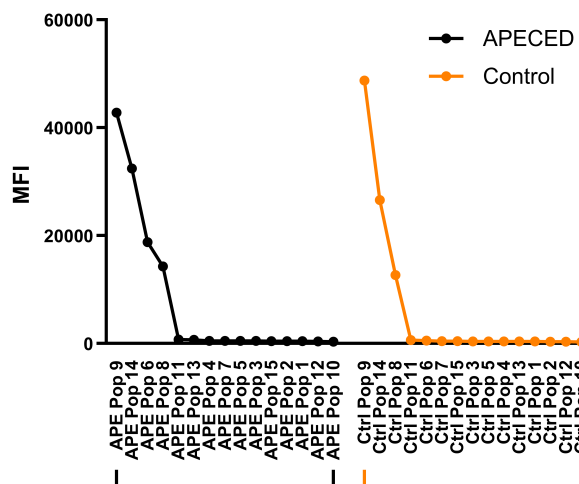

Supplement: Supplementary file 5 — Figure S1: Cell count, frequency of parent and median fluorescence intensity (MFI) per marker for each CD4+ T cell population detected. For each panel, the corresponding values are plotted in descending order for APECED patients and healthy controls separately, to better represent the population hierarchy relative to the markers analysed. [file SJI-104-e70134-s003.pdf]
